# Software engineering education in the era of conversational AI: current trends and future directions

**DOI:** 10.3389/frai.2024.1436350

**Published:** 2024-08-29

**Authors:** Cigdem Sengul, Rumyana Neykova, Giuseppe Destefanis

**Affiliations:** Computer Science Department, Brunel University London, Uxbridge, United Kingdom

**Keywords:** conversational AI, software engineering, computing education, literature review, LLM

## Abstract

The developments in conversational AI raised urgent questions about the future direction of many aspects of society, including computing education. The first reactions to the fast-paced evolution of conversational agents were varied: Some announced “the end of programming,” while others considered this “premature obituary of programming.” Some adopted a defensive approach to detecting the use of conversational AI and avoiding an increase in plagiarism, while others questioned, “So what if ChatGPT wrote it?” Nevertheless, questions arise about whether computing education in its current form will still be relevant and fit for purpose in the era of conversational AI. Recognizing these diverse reactions to the advent of conversational AI, this paper aims to contribute to the ongoing discourse by exploring the current state through three perspectives in a dedicated literature review: adoption of conversational AI in (1) software engineering education specifically and (2) computing education in general, and (3) a comparison with software engineering practice. Our results show a gap between software engineering practice and higher education in the pace of adoption and the areas of use and generally identify preliminary research on student experience, teaching, and learning tools for software engineering.

## 1 Introduction

With the advances in Large Language Models (LLMs), Generative AI (GenAI), and Conversational Agents (CAs), a wider discussion has started among computer scientists and educators, some urging for the necessity of “a major upheaval” in the field (Welsh, 2022), arguing the idea of writing computer programs and consequently, educating people for this purpose, “is headed for extinction.”

We are indeed at a transformative phase in artificial intelligence (AI) with Conversational AI (CAI), which, in this paper, is used to describe AI systems that interact with users in natural language, either through text or voice. This includes chatbots, virtual assistants, and AI-powered tools that understand and respond to queries, provide recommendations, or assist with tasks. We focus specifically on large language models (LLMs) and generative AI systems that produce human-like responses and content, such as OpenAI's ChatGPT (OpenAI, 2024), Google's Bard,^1^ and GitHub's Copilot (GitHub, 2024).

While CAI covers a wide range of applications, our analysis focuses on those relevant to software engineering practices and education. Welsh (2022) presents a provocative vision of the future, arguing that ‘most software, as we know it, will be replaced by AI systems that are trained rather than programmed.' While this perspective is debatable, it aligns with the current research trends observed in our study, where a significant portion of the literature examines the coding capabilities of CAI tools like ChatGPT. Similarly, computer science educators are exploring how these AI tools perform on course assignments and basic programming tasks. However, it is important to note that the field is still evolving, and the exact role of AI in future software development remains a topic of ongoing debate and research.

The paper aims to move beyond the current focus on introductory programming in the literature to software engineering education. Our goal is both to put into perspective the promising directions this new technology offers for improving the training of future software engineers, as well as the changes required to make the software engineering curriculum relevant to a software industry that is increasingly making use of these conversational tools. To this end, in this article, we seek to answer the following research questions:

**RQ1:** How is conversational AI currently influencing the software engineering industry?

**RQ2:** How is conversational AI impacting computing education?

**RQ3:** What do early experiences show in terms of promising improvements in educating future software engineers?

To answer these research questions, this paper reviewed the current status of CAI in computing and, more specifically, in software engineering education since 2018. The time span from 2018 to 2024 was selected to encompass a broad spectrum of work, ranging from the early stages of conversational agents to the recent developments in GenAI technology. Our paper confirms the scarcity of research on the impact of AI and LLMs in software engineering education in contrast to relatively extensive discussion on the general impact of GenAI, LLMs, and CAI in higher education (Finnie-Ansley et al., 2022; Yan et al., 2024). Therefore, there is a critical need for targeted research to explore how CAI can influence software engineering educational practices and ensure they keep pace with technological advancements. By identifying the key trends and open challenges in using CAIs in software requirement elicitation, design, development, testing, maintenance, and management activities, we highlight opportunities for aligning software engineering curricula and pedagogical approaches with the emerging needs of the AI-driven software industry.

The remainder of this article is structured as follows. Section 2 gives a brief overview of the existing reviews that consider the use of CAI agents in higher education and computing. Section 3 details the review and analysis methodology employed in the paper. Section 4 presents a quantitative analysis of the paper corpus, including a volume of publications, affiliated countries, collaborations, author networks, and research trends. Section 5 details the thematic analysis conducted to address the research questions posed in this study. Section 6 presents a discussion on the main findings and future directions, and Section 7 concludes the paper.

## 2 Background

Conversational agents, or chatbots, traditionally use Natural Language Processing (NLP) to respond to user queries in a dialog, mapping them to the best possible responses programmed into the system. Following the advances in AI, chatbots have increasingly adopted language models and deep learning, attempting to predict the likelihood of a sequence of words in a typical human interaction. The launch of OpenAI's ChatGPT (OpenAI, 2024) in 2022 demonstrated significant expansion to chatbot capabilities based on generative AI (GenAI). One of the most striking features of GenAI platforms and their use in conversational AI (CAI) is the adoption rate in a short period of time: launched on November 30, 2022, ChatGPT has been used by 1 million users in the first 5 days after its launch and reached 100 million users in its first 2 months (Dwivedi et al., 2023). Since then, the development of LLMs has accelerated significantly, especially with the release of commercial products from major tech companies. For instance, Google released Gemini (see text footnote ^1^), Meta rolled out LLaMA (Touvron et al., 2023), and Anthropic introduced Claude.^2^ Many other specialized models designed for specific tasks have emerged, showcasing a variety of architectures and functionalities in diverse domains (Hou et al., 2023).

The literature on the use of CAI in higher education predominantly focuses on general education rather than specific applications within software engineering. Okonkwo and Ade-Ibijola (2021) present a systematic review of the use of chatbots in education prior to the release of ChatGPT, which highlights their ability to provide personalized help quickly and identifies integration challenges and opportunities. Memarian and Doleck (2023) explore the use of ChatGPT in education, providing a thematic analysis that reveals its potential for personalized learning and complex teaching activities but also notes significant issues such as plagiarism and the need for safeguards to protect academic integrity.

Jürgen Rudolph and Samson (2023) and Baidoo-Anu and Owusu Ansah (2023) explore the implications of ChatGPT integration in terms of assessment and learning. The former discusses how the evolution from a non-profit to a commercial model by OpenAI affects the deployment and development of such AI technologies. The conclusion from the articles is that while ChatGPT offers extensive benefits such as improved engagement and personalized learning experiences, it also necessitates careful consideration of ethical standards, privacy issues, and potential biases in AI training. Furthering this discussion, Yan et al. (2024) conduct a systematic scoping review and highlight the ethical and practical challenges in employing LLMs for educational tasks such as feedback provision and content generation, recommending strategies for ethical integration and the adoption of human-centered approaches in the development of AI educational tools.

Several studies, such as those by Chen S. et al. (2023) and Lo (2023), examine the roles and impact of ChatGPT's potential to revolutionize virtual teaching assistants and intelligent tutoring systems. They emphasize the need for educators to understand the implications of this technology and adapt their teaching practices accordingly.

In the field of computing education, only a few papers have surveyed the literature on LLMs and their implications for software engineering education. Neumann et al. (2023) conduct a rapid gray literature review of papers published up to January 2023 and present challenges and opportunities that emerge from the release of ChatGPT. However, their review is limited in scope, with only a few papers discussed. On the other hand, Finnie-Ansley et al. (2022) present a working group report on GenAI in computing education. The report includes a comprehensive literature review, with a corpus of papers up to August 2023. The authors also incorporate survey findings, insights from interviews with students and teachers, and ethical considerations related to the use of GenAI in computing education. Furthermore, they benchmark the performance of current GenAI models and tools on various computing education datasets, offering a practical assessment of their capabilities.

All of the above works highlight the transformative potential of ChatGPT in reshaping teaching, learning, and assessment practices. However, they largely omit detailed discussions on the unique requirements of and impact on software engineering education. The only exception is Finnie-Ansley et al. (2022), which argues for the effective integration of LLM technologies in computing education, yet they stop short of going deeper into software engineering specifics and primarily consider coding-related tasks.

In contrast, there is a growing body of research assessing the capabilities and limitations of LLMs for various tasks across the software development lifecycle (SDLC). Among these are several survey papers on GenAI in software engineering, which span a wide range of depth, scope, and methodology. Hou et al. (2023) conduct a systematic literature review, analyzing 395 research papers from January 2017 to January 2024, to categorize LLM applications in Software Engineering (SE), examine data methodologies, and evaluate performance optimization strategies and effectiveness across SE tasks. Santhanam et al. (2022) conduct a systematic mapping study, reviewing research articles to categorize the applications of AI bots in SE. Del Carpio and Angarita (2023) present a systematic analysis of different assistant solutions, including recommendation systems and chatbots for SE tasks. Liang et al. (2024) present insights from 410 developers to examine the usability of AI programming assistants like GitHub Copilot (GitHub, 2024), identifying key motivators and barriers in their adoption. Fan et al. (2023) and Belzner et al. (2024) provide comprehensive reviews and case studies on the role of LLMs across the entire SE process, emphasizing the need for hybrid techniques that combine traditional SE methods with AI-driven approaches.

These studies highlight the increased interest and ongoing challenges in integrating AI into software engineering. However, their primary focus is on synthesizing findings and implications for the software engineering industry, not necessarily for software engineering education. This paper aims to bridge this gap by analyzing the literature on practice and education-oriented papers through the lens of their implications for software engineering education.

## 3 Methodology

### 3.1 Review methodology

**Figure 1:**
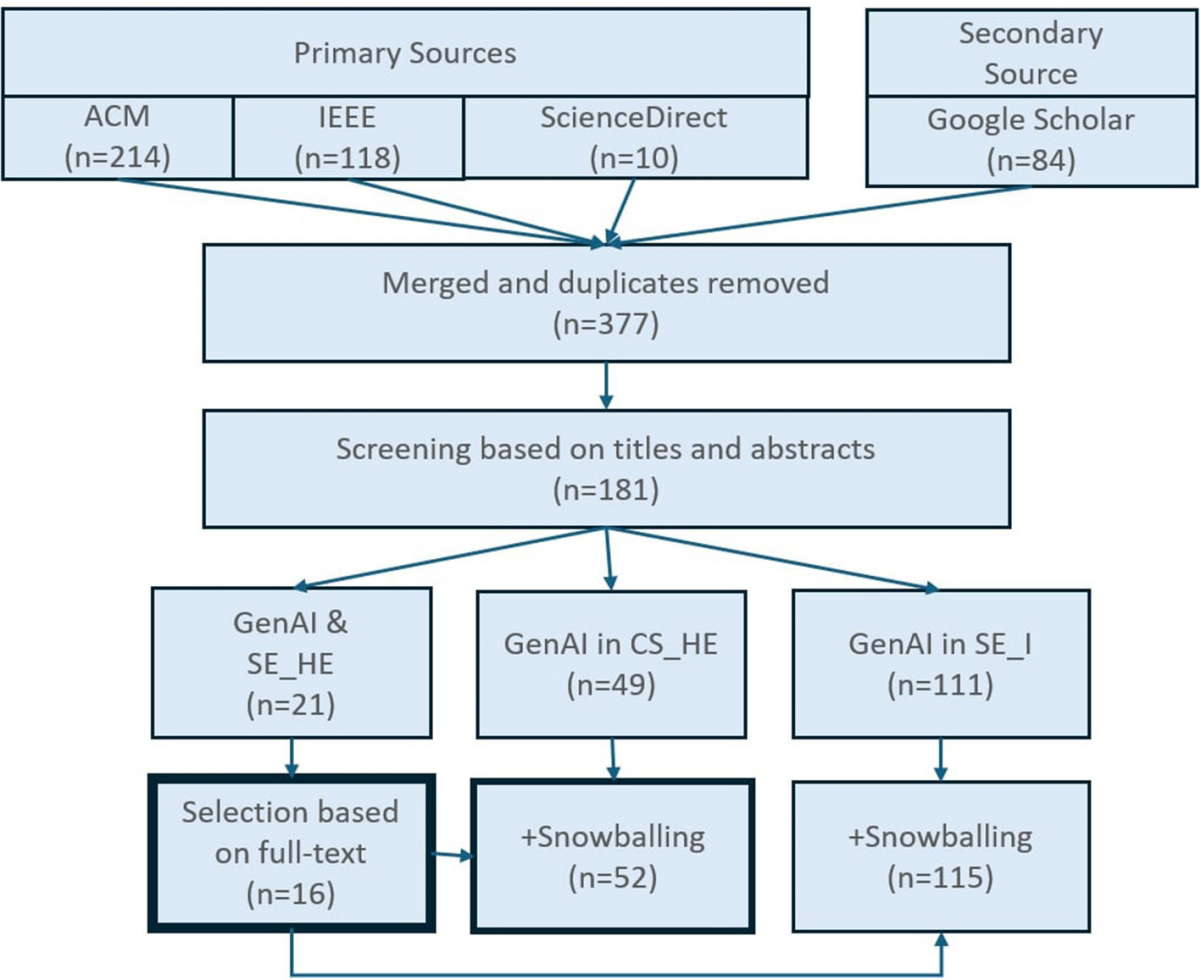
A PRISMA-guided process was followed for selecting studies for inclusion in the review. The primary sources were ACM, IEEE, and Science Direct databases, and as a secondary source, Google Scholar was used. The initial number of records identified was 426, and 377 when duplicates were removed. After screening the paper titles and abstracts based on the exclusion criteria, 183 papers were included in the study and categorized into three categories: HE_SE, CS_HE, and SE_I. For SE_HE, 16 were selected based on full text. For CS_HE and SE_I, 52 and 115 article abstracts were included at the end in the qualitative and quantitative analysis.

A rapid review approach was used, and a PRISMA-guided (Preferred Reporting Items for Systematic Reviews and Meta-Analysis; Moher et al., 2009) process was followed to select studies for inclusion in the review. The diagram of the search strategy is depicted in Figure 1. The search process used three main sources: IEEE Explorer, ACM Digital Library, and ScienceDirect. Google Scholar was used as an additional complementary resource that mainly helped capture recent contributions published in arXiv. All arXiv research that was later published as a peer-reviewed article was included in this review, but the rest was not included. The only exceptional addition is Chen E. et al. (2023), which is an open-source tool hosted elsewhere.

**Search string:** The keywords used in the search string include:


        Conversational OR Conversational AI OR
        prompt engineering OR code assistance
        OR pair program OR coPilot OR chatGPT
        OR Bard
          AND
        Software Systems OR Software Engineering
        OR Systems Development OR Computer
        Science Education OR Engineering
        Education


The search strategy included two main components. The first subset aims to capture work related to conversational AI agents, and after a few tries, we decided to include specific examples like ChatGPT, Bard, and coPilot to ensure good coverage. The second subset limits our search to either software engineering practice or education or computing education papers.

The meta-data extracted from the search include the source and full reference, which includes the authors and their institutions, the country where it is situated, author keywords, and the abstract.

**Inclusion and exclusion criteria:** For screening and eligibility checks, we have established inclusion and exclusion criteria. The articles that mainly report on methods to improve a particular conversational agent technology were excluded.

Papers that are not written in English and do not present research findings, e.g., 1–2 page abstracts, workshop proposals, and pure opinion papers, were also excluded.

The research aimed to explore research articles on the contributions of conversational agents to software engineering and its education. After excluding papers based on the exclusion criteria, papers focusing on experiences, trials, and solutions that utilize conversational agents in the following three categories were included in the study:

- Software engineering in practice, denoted as SE_I
- Computer science higher education, denoted as CS_HE
- Software engineering in higher education, denoted as SE_HE

It must be noted that studies emphasizing solely the coding aspect of software engineering were excluded from SE_HE category. This decision stems from the recognition that software engineering has a broader scope and involves not only coding but other aspects, such as software requirements, design, development, testing, and management. These studies were considered part of CS_HE category. CS_HE excludes purely opinion papers if they do not report on actual experiences, trials, and solutions that utilize conversational agents in a computing education setting.

**Paper selection:** In the preliminary selection process, the first author evaluated all papers returned based on the title and the abstract and classified them as included or excluded based on the inclusion and exclusion criteria. Then, the second and third authors verified the excluded papers and justifications and the included papers and their extractions.

The initial number of records identified was 426, and 377 when duplicates were removed. After screening the paper titles and abstracts based on the exclusion criteria, 183 papers were included in the study and categorized into three distinct categories: SE_HE, CS_HE, an SE_I. For SE_HE, 16 were selected based on full text. For CS_HE and SE_I, 52 and 115 article abstracts were included at the end in the quantitative and qualitative analysis.

### 3.2 Thematic analysis methodology

We have used a mixed methodology as the number of papers in each category was significantly different. We also aimed for a deeper analysis for SE_HE category and reviewed full papers, while for SE_I and CS_HE, we only reviewed titles, keywords, and abstracts.

For the papers in SE_I, we conducted a deductive thematic analysis using Claude3 (see text footnote ^2^). We provided the title, abstract, and author keywords to Claude3 and tasked it with classifying the papers and assigning them to categories and subcategories. We have categorized the papers following the six phases of the Software Development Life Cycle (i.e., requirements engineering, software design, software development, software quality assurance, software maintenance, and software management). For this study, including author keywords was essential. As experts in their respective fields, the authors have the most comprehensive understanding of their work's main topics and themes, so this expert input was considered valuable for a meaningful thematic analysis. To ensure the reliability of classifications, two authors independently reviewed the results provided by Claude3, compared their classifications, and discussed any discrepancies to reach a consensus on the final categorization. While no factual inaccuracies were found, in a few cases, additional information was added when the summary was too generic and had not captured essential details from the abstract. This process helped refine the automated classifications and ensure they accurately reflected the abstracts' content. The results of this analysis are presented in Section 5.1.

For the papers in CS_HE category, we conducted a reflexive thematic analysis (RTA; Braun and Clarke, 2023) of the abstracts with ChatGPT4.0 acting as the pair coder. It must be noted that we have not tried to achieve consistency in the use of Claude3 and ChatGPT4.0, as we have used these tools mainly for guidance and manually reviewed and refined outputs. Our ability to identify what we saw in the data was informed by existing concepts, our own knowledge of the literature, and the convention of academic abstracts. Hence, while the analysis was dominantly inductive, a degree of deductive analysis was employed to ensure that the open coding produced themes that were meaningful to the research questions.

More specifically, the analysis was carried out following the six phases of RTA:

- **Familiarization:** We began by thoroughly reading all the abstracts to familiarize ourselves with their content and context.
- **Generation of initial codes:** To perform the initial coding of the abstracts, we asked ChatGPT4.0 to perform a thematic analysis in addition to manually generated codes. One author refined all produced themes and codes, and all authors sense-checked ideas by exploring multiple assumptions, interpretations, and meanings of the data, following a collaborative and reflexive approach.
- **Generating themes:** From these initial codes, we developed themes that represented the major concepts and ideas found in the data. This involved identifying patterns and relationships among the codes to form coherent themes.
- **Review of themes:** All authors participated in reviewing the themes to ensure they were consistent and relevant. This iterative process allowed us to refine the themes and ensure they accurately represented the data.
- **Definition of themes:** The definitions and names for each theme were established, reflecting the underlying data and authors' interpretations.
- **Reporting:** Section 5.2 presents the detailed results of this analysis, outlining our findings and the thematic map developed from the abstracts.

By using RTA with LLM-based tools and a detailed manual review process, we ensured a reliable examination of the abstracts. The reflexive approach helped us revisit our assumptions and interpretations, making sure the identified themes truly reflected the data and were relevant to our research questions.

Finally, for the papers that fall under SE_HE, we have read the full papers and categorized the work based on the Software Development Lifecycle and the Guide to the Software Engineering Body of Knowledge (SWEBOK; Bourque and Fairley, 2014), which describes generally accepted knowledge about software engineering. We have also, as part of the analysis, extracted a summary, including the main research questions, methodology, and findings guided by the questions below:

- Which sub-area of software engineering, if any, does the paper focus on?—Answers vary from paper to paper and may include software development, software testing, and requirements engineering, among others.
- Does the study target a specific group of people? Which groups?—Possible answers are (a) No specific group, (b) educators, (c) students, and (d) professional developers.
- Which conversational agent technology is used in the study? What is the role of the conversational agent?—Answers vary from paper to paper and include new technology created for the paper or more recent AI-based conversational agents like ChatGPT, Bard, and the like. Conversational agents may be used for code generation, providing explanations, or merely for comparison to student-generated work.
- What is the study method used?—Answers vary from paper to paper. The goal is to extract the attributes of each method, e.g., experimentation with a conversational agent, a user study involving a questionnaire, interview, or prototype controlled experiments, among others.

The findings of this analysis are presented in Section 5.3.

## 4 Corpus analysis

### 4.1 Publication years and countries

**Figure 2:**
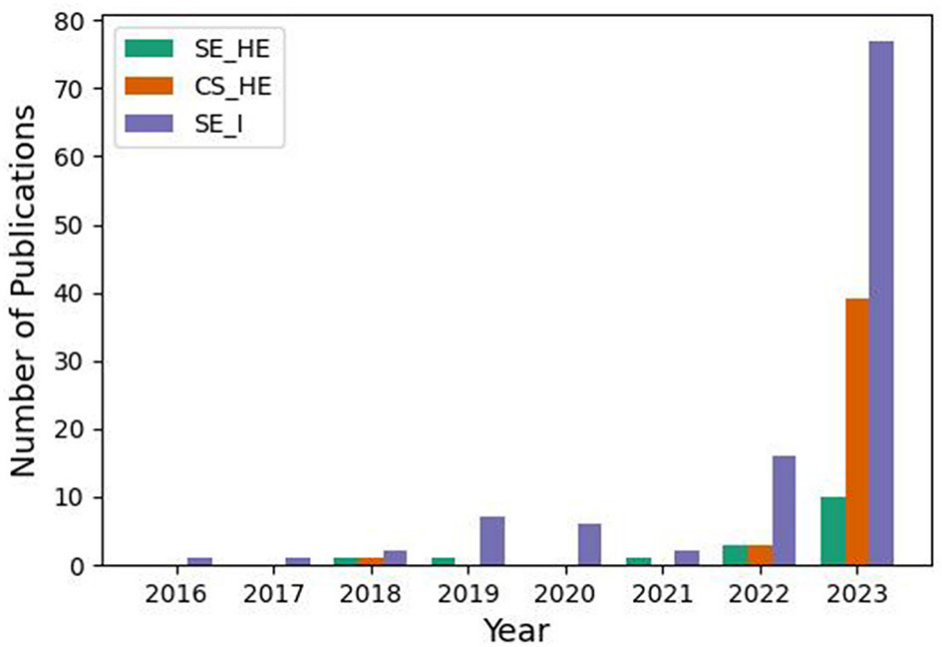
The number of publications per year showing an exponential increase in conversational agents research in (1) SE education in HE, (2) CS education in HE, and (3) SE practice, albeit prominently in the 3rd category.

Extracted papers were published between the years 2018 and 2024 (January). Figure 2 illustrates a sharp increase in the number of publications from 2018 to 2023 for each category: (1) SE_I (Software Engineering Industry), (2) CS_HE (Computer Science Higher Education), and (3) SE_HE (Software Engineering in Higher Education). However, the increase is most prominent in SE_I, and the number of publications for SE_HE is still low compared to the rest. We expect the same trend to continue in 2024.

**Figure 3:**
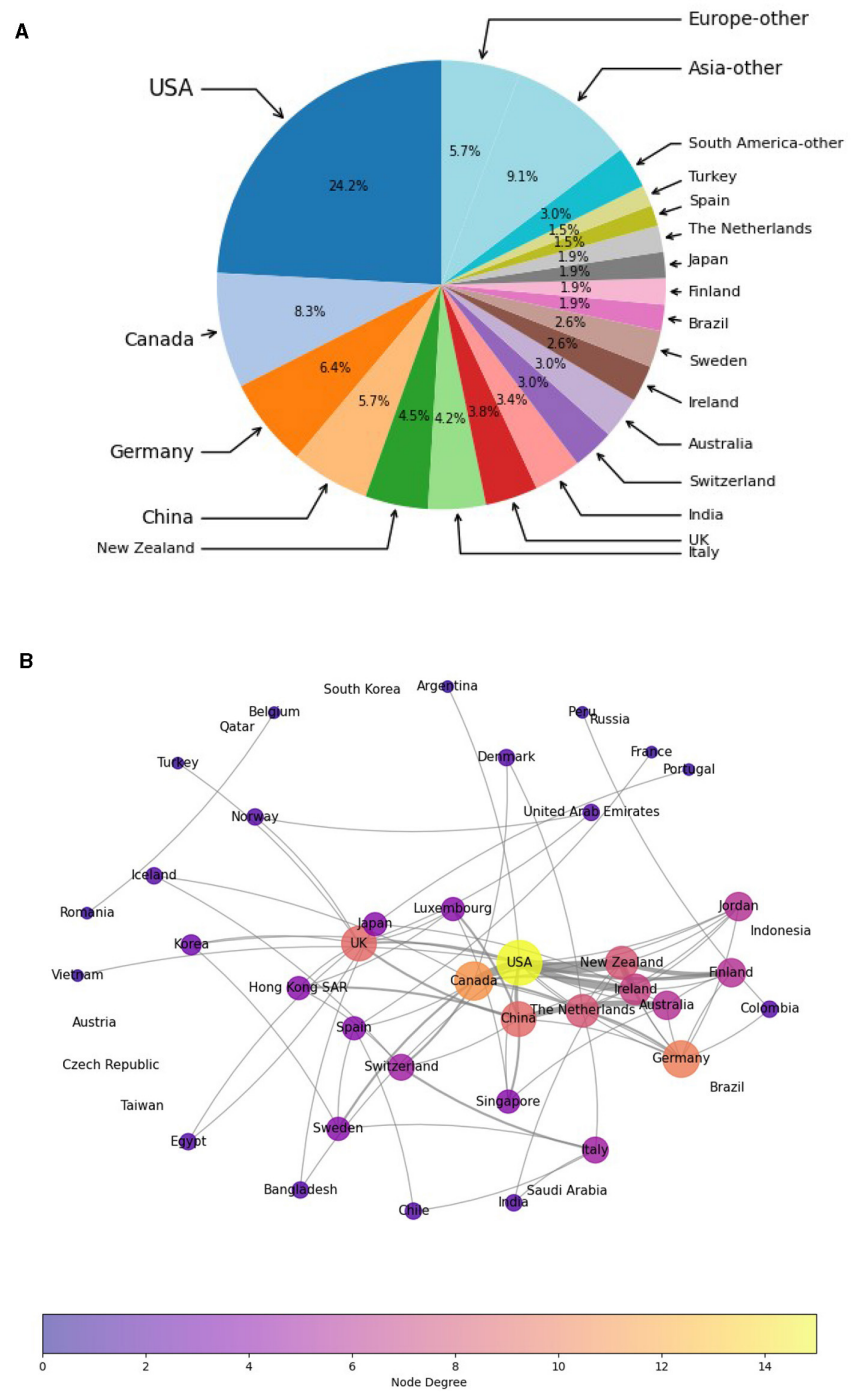
Which countries publish most in this area, and how do they collaborate with other countries? **(a)** Country distribution of publications in all categories. **(b)** Countries in collaboration.

Figure 3A shows most publications are by authors affiliated with an institution in the USA. Canada, Germany, and China also have a strong presence. Nevertheless, research in this area spans many countries in Europe, Asia, North America, and South America, showing a high diversity of studies, including evaluating CAI in languages other than English.

The authors from different countries are also in close collaboration; Figure 3B shows strong links between the USA and Canada, China and Australia, Germany and Netherlands, and collaborations among New Zealand, Ireland, Finland, and the USA. These results confirm the existence of already robust international collaborative networks in this area.

### 4.2 Author networks

**Figure 4:**
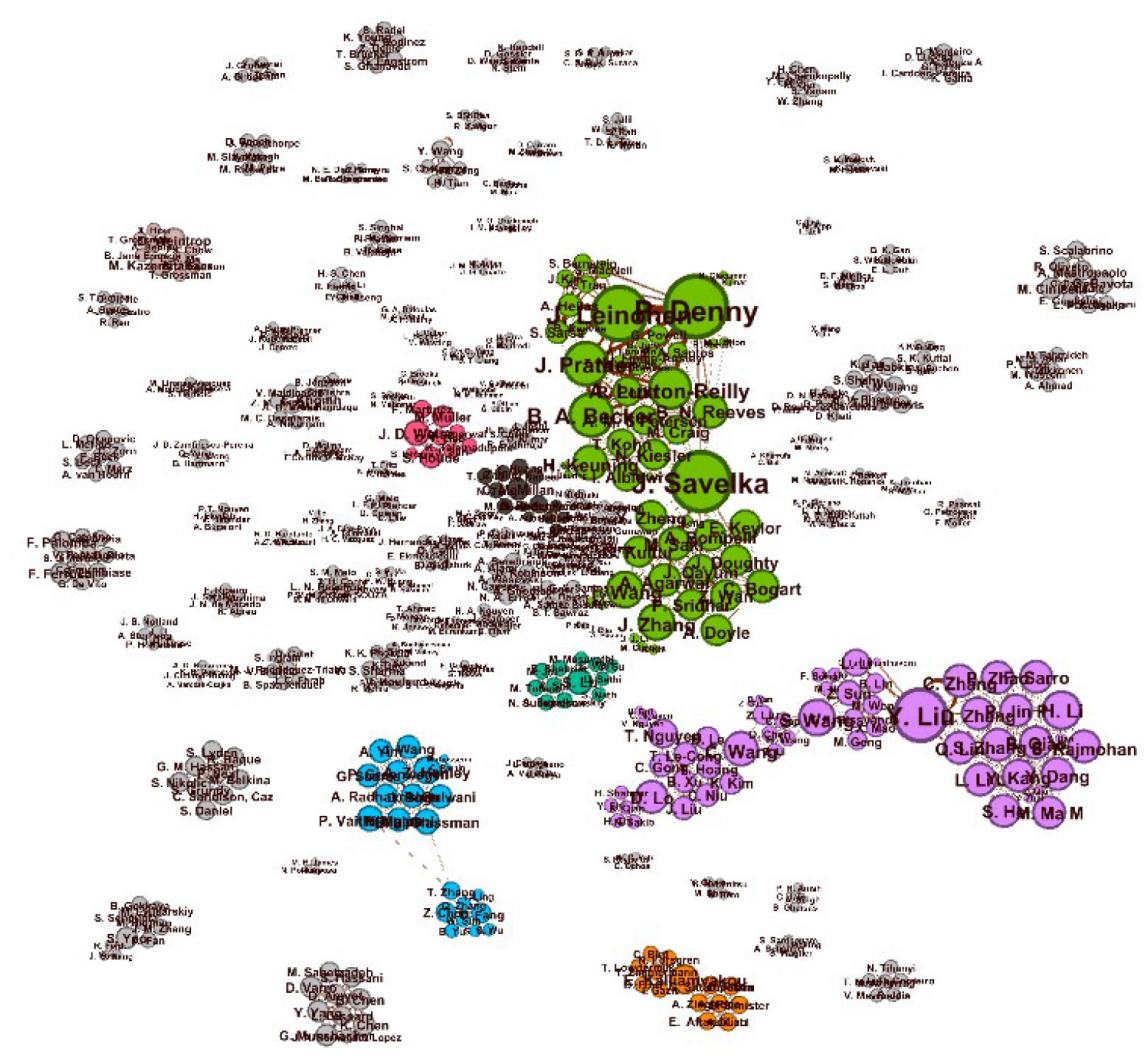
Author clusters generated by Force Atlas2 algorithm using the Gephi software. The connected components vary in size. The weighted edges indicate the number of collaborations between authors, i.e., the thicker the edges, the more the two authors collaborated.

To further understand collaboration communities, we analyzed the co-author networks, where each node is an author, and each edge between two nodes indicates a collaboration on a paper. Figure 4 presents the resulting network, laid out according to the Force Atlas 2 algorithm in the Gephi Visualization Software (Bastian et al., 2009). Key authors, or those central to the network with many connections, can be seen as larger nodes, often positioned toward the center of the network clusters. The edge weight highlights the number of common publications for each author pair. The figure shows a number of key authors (larger nodes) and a few large communities of collaborations but a significant number of smaller, isolated groups or pairs of collaborators, indicating a growing interest in the area.

### 4.3 Research trends

To spot rising or declining trends in research topics over time, we used the author keywords derived from the corpus of the 183 publications in all categories. When author keywords were absent, we used the publisher keywords, with the limitation that these words were sometimes too generic, e.g., “codes” and “visualization.” Finally, in rare cases when neither author nor publisher keywords were present, we tokenized the title to create the author keywords. As this exercise resulted in many keywords, we have grouped the majority of the keywords as shown in Appendix Table 1 and eliminated the ones that did not fit in any group and had low occurrence.

**Figure 5:**
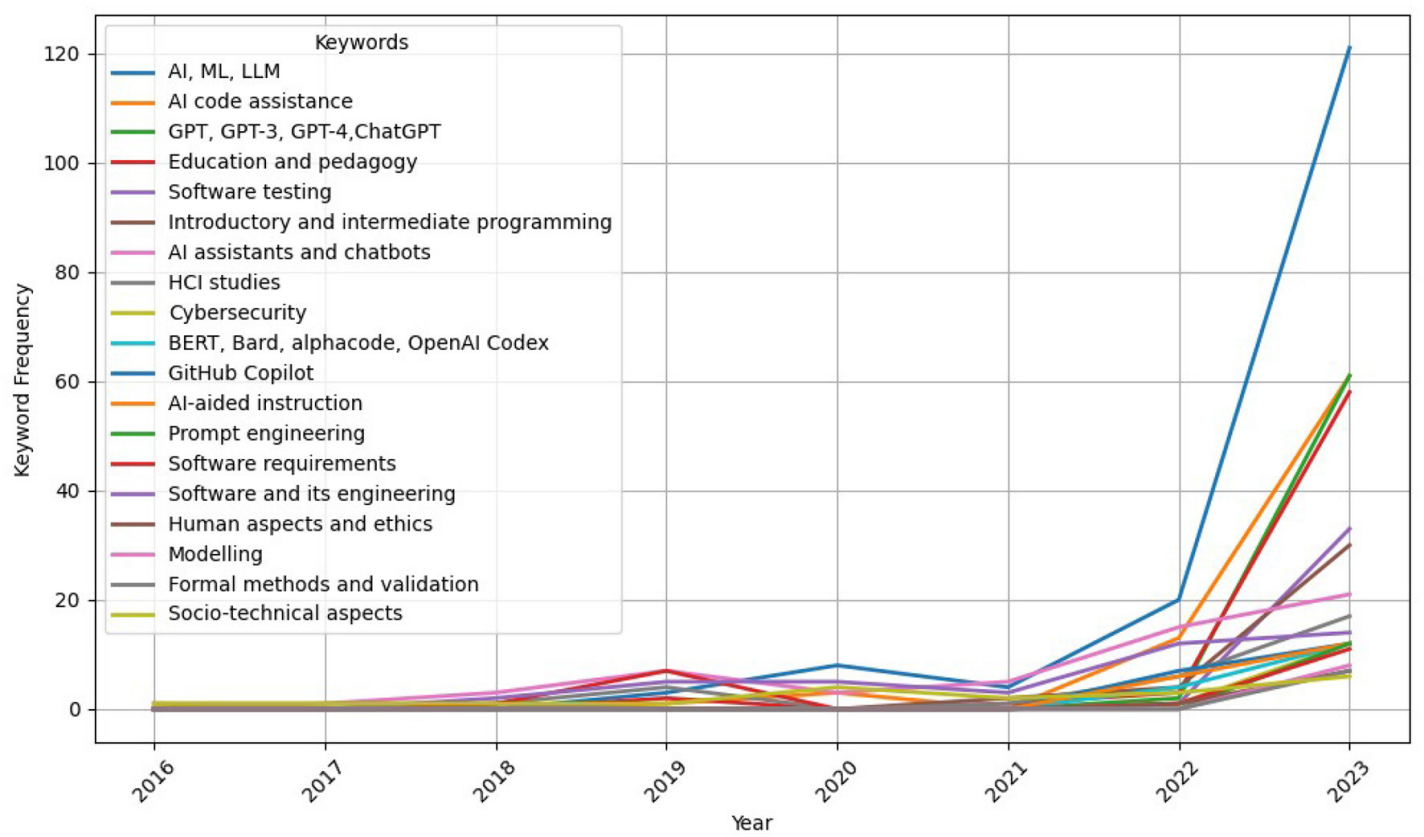
The yearly frequency of terms in Appendix Table 1. Each line indicates how the prominence of these topics has changed, highlighting the prominent focus on “AI, ML, LLM,” and emerging interest in “prompt engineering,” emphasizing the growing research emphasis on using this technology in “education and pedagogy,” and “introductory and intermediate programming.”

Figure 5 tracks the yearly frequency of these term groups from Appendix Table 1 such as “AI assistants and chatbots,” “education and pedagogy,” and “software and its engineering.” Each line indicates how the prominence of these topics has changed, highlighting the focus on “AI, ML, LLM,” and emerging interest in “prompt engineering,” and the growing research emphasis on using this technology in “education and pedagogy,” and “introductory and intermediate programming.”

To dig deeper and identify clusters of research topics and their interconnections within the categories, we next carried out a term co-occurrence analysis using the author keywords. The co-occurrence network graph presents the interconnectedness and relative frequency of terms extracted from author keywords in our corpus. Each node symbolizes a unique term, and larger nodes indicate higher occurrence frequencies. The node's color indicates the number of connections, i.e., its degree.

**Figure 6:**
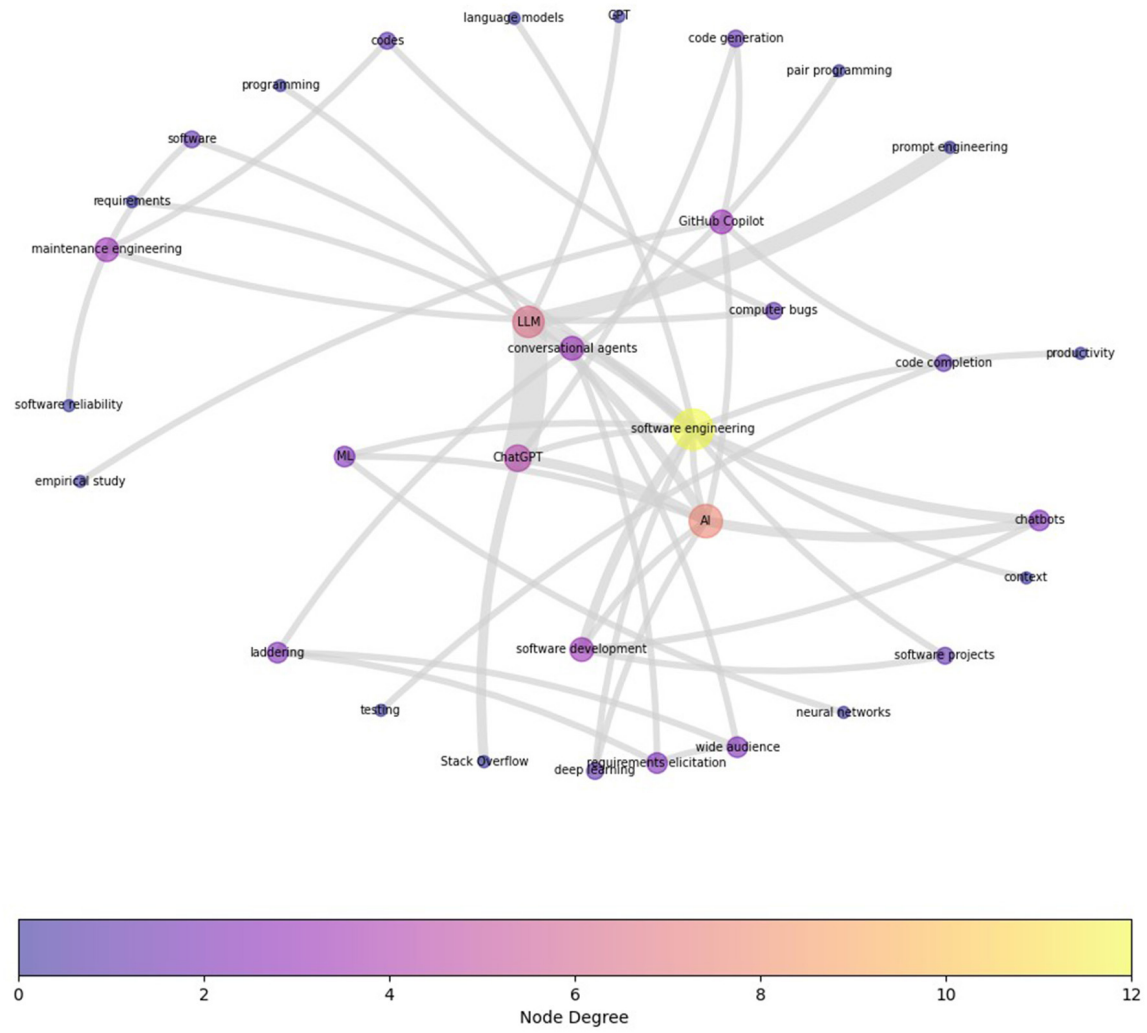
Term Co-occurrence Map for HE_I category based on 115 peer-reviewed articles in this category. To see the prominent clusters more clearly, the graph is reduced to omit all edges (nodes) that have an edge weight of less than two (i.e., the co-occurrence needs to exist in more than one publication). Notable clusters are around the current generative AI conversational agents, e.g., “ChatGPT,” and “Github Copilot.” While there is a focus on “software development,” the emphasis on “prompt engineering” and “maintenance engineering” is also apparent.

In all categories, expectedly, the most visible clusters are around “LLM,” “artificial intelligence (AI),” “chatbots,” “software,” and “software engineering.” Figure 6 depicts the term co-occurrence map for SE_I category based on 115 peer-reviewed articles. To see the prominent clusters more clearly, the original co-occurrence graph was filtered to omit all edges that have an edge weight of less than two (i.e., the co-occurrence needs to exist in more than one publication). Notable clusters are around “ChatGPT” and “Github Copilot;” however, there are more notable smaller clusters, especially around “prompt engineering” and “maintenance engineering.”

**Figure 7:**
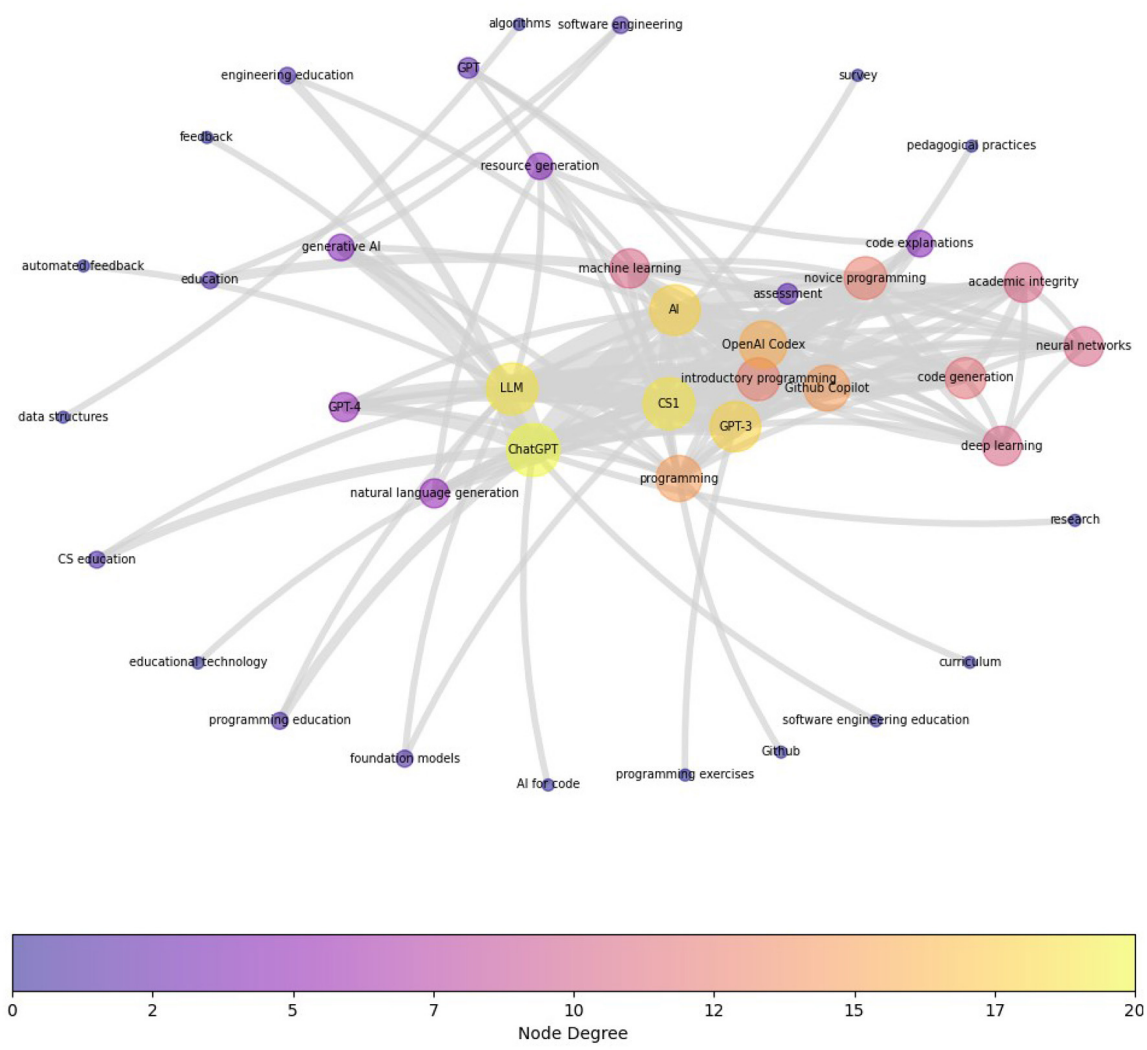
Term Co-occurrence Map for HE_CS category based on 52 peer-reviewed articles in this category. To see the prominent clusters more clearly, the graph is reduced to omit all edges (nodes) that have an edge weight of less than two (i.e., the co-occurrence needs to exist in more than one publication). Notable clusters are around the current generative AI conversational agents e.g., “ChatGPT”, “OpenAI Codex,” “GPT-3”, showing the community is mainly focused on testing the utility of these tools. Other main areas of current interest are “CS1/introductory/novice programming,” and “academic integrity.”

Figure 7 shows the term co-occurrence map for HE_CS category based on 52 peer-reviewed articles in this category. Again, to see the prominent clusters more clearly, the graph is reduced to omit all edges that have an edge weight of less than two (i.e., the co-occurrence needs to exist in more than one publication). Notable clusters are around the current generative AI conversational agents e.g., “ChatGPT,” “OpenAI Codex,” “GPT-3,” showing the community is mainly focused on testing the utility of these tools. Other main areas of current interest are “CS1/introductory/novice programming,” and “academic integrity.” Current integration efforts are channeled understandably on introductory programming, which is aligned with the level of complexity the current GenAI tools are able to handle. The focus on academic integrity underlines the reactionary reception of these tools in computer science education.

**Figure 8:**
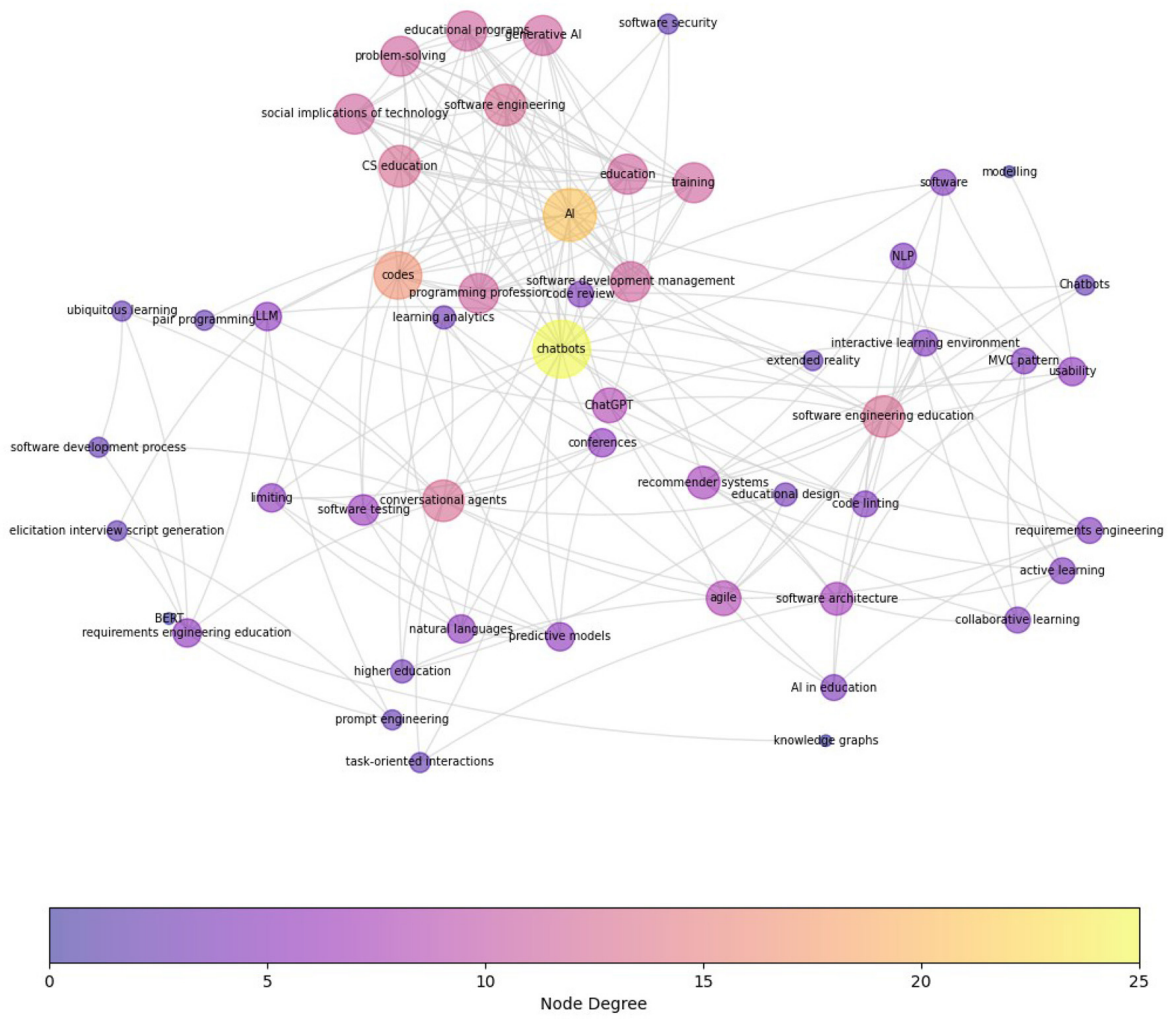
Term Co-occurrence Map for HE_SE category. This map presents the interconnectedness and relative frequency of terms extracted from a corpus of over 16 peer-reviewed articles in HE_SE category. Each node symbolizes a unique term, where larger nodes indicate higher occurrence frequencies. Compared to previous co-occurrence graphs, this graph hasn't been reduced due to its smaller size. Notable smaller clusters are around “software development management”, “agile,” “educational design,” and “software architecture,” and “programming profession”, which highlight key focal areas of recent research.

Finally, Figure 8 presents the term co-occurrence map for the HE_SE category extracted from 16 articles in HE_SE category. While this figure is more sparse than other categories, “software development management,” “agile,” “educational design,” and “software architecture,” and “programming profession” appear as the main software engineering areas besides the aforementioned clusters. In addition, “ChatGPT” and “training” have emerged as focal areas of recent research.

In the next section, we explore these trends more deeply by conducting a thematic analysis of each category, with the goal of answering the research questions we set out in the introduction.

## 5 Thematic analysis findings on GenAI in software industry and education

### 5.1 RQ1—how is AI shaping the software industry?

In categorizing papers within the Software Engineering (SE_I) category, we have adopted a framework based on the six phases of the Software Development Life Cycle (SDLC). Our categorization encompasses the following phases: requirements engineering, software design, software development, software quality assurance, software maintenance, and software management. This approach aligns with and expands upon the activities in software processes as described by Sommerville (2015) and the core knowledge areas defined in SWEBOK (Bourque and Fairley, 2014). While our categorization closely mirrors these established frameworks, we have made slight adjustments in terminology and scope to better reflect the integration of AI-assisted techniques and current industry practices in software engineering.

**Figure 9:**
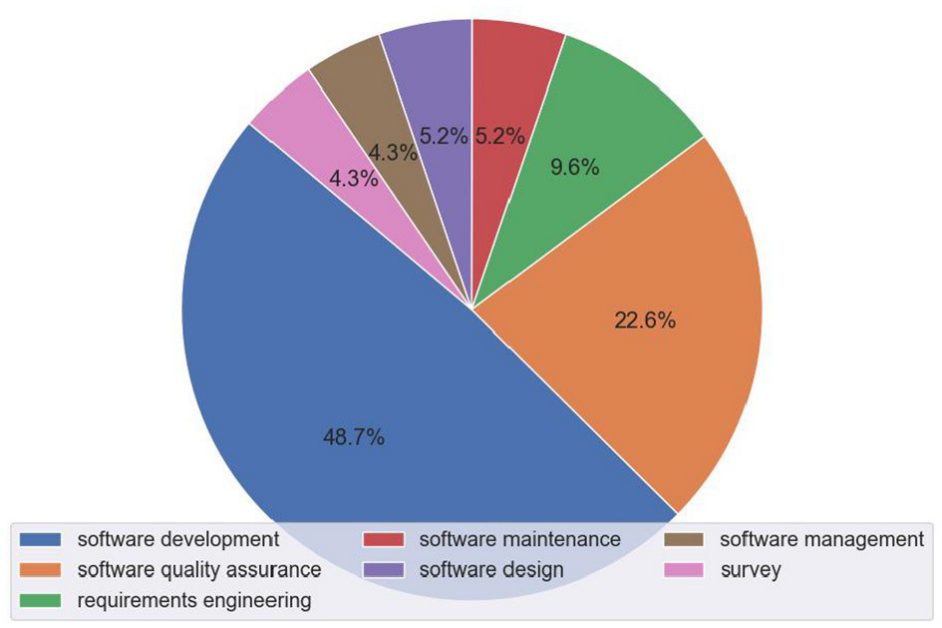
Distribution of papers per SE domain.

**Figure 10:**
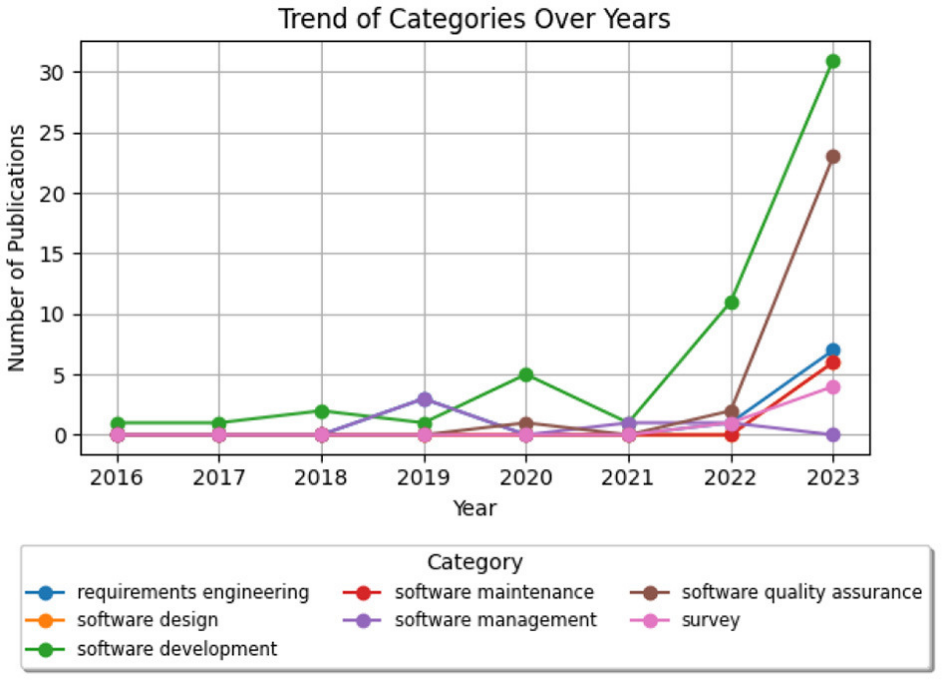
Papers per SE domain over the years.

Figure 9 illustrates the distribution of research papers across the different SE categories, with software development having the largest share at 48.7%, followed by software quality assurance at 22.6%. Figure 10 shows the trend of papers published in each SE category over the years, indicating a rapid growth in applications across all areas, but especially in software development and software quality assurance. Within each category, we have identified specific subcategories, i.e., the specific SE tasks that have been the focus of Conversational AI research. Table 1^3^ lists the identified subcategories and the respective number of papers for each subcategory. It is important to acknowledge that the boundaries between software engineering tasks are often blurred, and many studies assess or cover multiple aspects concurrently. For instance, the research on program synthesis, evaluating code quality, performance analysis, and bug detection can be considered relevant to both software development and software quality assurance. In such cases, we reviewed the article to reach a consensus on the assigned subcategory.

**Table 1:** Distribution of the number of papers from SE_I category across the six phases of software development lifecycle.

**SE activity**	**SE task**	
Requirements engineering (11)	Ethics (1)	Goal-oriented modeling (1)
	Requirements analysis (3)	Requirements elicitation (4)
	User stories (1)	Requirements classification (1)
Software development (56)	Program synthesis (26)	Api recommendations(1)
	Code completion (2)	Code transpilation (1)
	Human aspects (3)	Code understanding (4)
	Digital coworker (4)	Software process (1)
	Explainability (1)	Digital mentor (2)
	Program repair (1)	Pair programming (1)
	Other (2)	Development tools (6)
	Dataset (1)	
Software quality assurance (26)	Code efficiency (1)	Dataset (1)
	Code reviews and documentation (1)	Vulnerabilities detection (3)
	Load testing (1)	Program repair (8)
	Security & reliability (4)	Testing automation (7)
Software design (6)	Gui (1)	Model-driven engineering (1)
	Use cases (1)	System architecture (1)
	Modeling (2)	
Software maintenance (6)	Code reviews (1)	Q&A bots (2)
	Log parsing (1)	Code summarization (1)
	Traceability (1)	
Software management (5)	Community smell (1)	Agile project management (1)
	Expert recommendation (1)	Effort estimation (1)
	Software process improvement (1)	
Survey (5)	Systematic review (1)	Mapping study (1)
	Other (3)	

Conversational AI technology is still in its infancy, but its broad implications in software engineering are evident. While many studies focus on code generation and repair, where LLMs have outperformed novice developers and accelerated routine tasks for experts, research now extends to less explored areas such as team training, expert recommendations, documentation, and maintenance. The benefits of applying LLMs vary across different areas of the SDLC, but the prevailing trend involves integrating AI at varying levels while maintaining human oversight to address limitations.

The following subsections lay out the key trends, promising results, and challenges for each software engineering category based on the findings from the surveyed papers.

**Requirements engineering** covers the elicitation, analysis, specification, and validation of software requirements and the management of these requirements during the software product lifecycle (Bourque and Fairley, 2014). Recent research reveals several key insights into the application of AI technologies in RE processes. Conversational agents can effectively assist in requirements elicitation, capturing diverse stakeholder needs, as evidenced by studies on systems like LadderBot (Rietz, 2019; Rietz and Maedche, 2019). LLMs demonstrate potential for automatically extracting domain models from natural language requirements documents (Arulmohan et al., 2023). AI-generated user stories can also facilitate the integration of human values into requirements, serving as creative prompts for stakeholders (Marczak-Czajka and Cleland-Huang, 2023). Regarding the quality of AI-generated requirements, Ronanki et al. (2023) found ChatGPT-generated requirements to be highly abstract, atomic, consistent, correct, and understandable. The same researchers, however, also emphasized the need for AI-centric Requirements Engineering (RE) frameworks that incorporate ethics and trustworthiness. Collectively, these studies demonstrate AI's potential in enhancing RE processes while highlighting the continued importance of human expertise for ensuring the validity and applicability of generated requirements.

**Software design** refers to creating detailed specifications and blueprints for the software system, defining its architecture, components, interfaces, and data flow, which serve as a guide for the development and implementation stages. AI is showing significant potential in generating software designs from requirements. Interactive dialogues with LLMs, help elaborate design goals and constraints, suggesting design artifacts ranging from UML (Unified Modeling Language) models to user interface layouts, enhancing both productivity and design quality (Ahmad et al., 2023; De Vito et al., 2023). However, ensuring the consistency and completeness of these machine-generated designs remains a challenge particularly when integrating design information across different notations and abstraction levels (Cámara et al., 2023; Chen K. et al., 2023). AI design assistants offer promising advancements (Ahmad et al., 2023; Brie et al., 2023); however, they also require a deep understanding of their capabilities and limitations from designers. Human input remains crucial for evaluating and refining generated designs. An emerging trend is the importance of prompt engineering techniques, becoming essential for guiding AI toward more relevant and coherent results, improving the integration and usability of AI tools in design processes (De Vito et al., 2023).

**Software development**, which encompasses the implementation activities of SDLC, has seen the most rapid adoption of AI coding assistants. Models like Codex and Copilot can generate code from natural language descriptions, autocomplete partial programs, and even explain and translate code. AI assistants help developers handle routine coding tasks and offer relevant on-demand suggestions (Storey and Zagalsky, 2016). Studies show these tools can significantly boost developer productivity, especially for less experienced programmers (Moroz et al., 2022; Nguyen and Nadi, 2022). However, the generated code still falls short of expert human-written code in terms of best coding practices, robustness, and maintainability (Nguyen and Nadi, 2022; Moradi Dakhel et al., 2023).

Researchers have identified several challenges in the integration of generative AI in software development. These include the need to customize AI models to individual developers' knowledge and project contexts for more relevant suggestions (Moroz et al., 2022), the importance of developers acquiring skills in prompt engineering and code inspection (Sun et al., 2022), and the ongoing challenge of integrating AI assistants into existing software engineering workflows and development environments (Barke et al., 2023). These challenges highlight the complex interplay between AI capabilities and human expertise in the evolving landscape of software development.

**Software quality assurance** refers to the systematic processes and activities designed to ensure that software meets specified requirements and quality standards. This involves the implementation of various practices such as code reviews, testing, and audits to identify defects and ensure the reliability, efficiency, and security of the software. In quality assurance, AI is being applied to generate test cases (Guilherme and Vincenzi, 2023), reproduce bug reports (Kang et al., 2023), localize faults (Li H. et al., 2023), and suggest code patches (Xia et al., 2023). LLMs can use requirements specifications and code context to generate relevant test scenarios and oracles (Okanović et al., 2020; Brie et al., 2023). By analyzing bug reports and comparing code versions, they can often pinpoint the root cause of errors and even suggest fixes (Tony et al., 2022; Dantas et al., 2023). AI-based static code analyzers are also improving in their ability to pinpoint style issues and spot potential bugs and security flaws (Pan and Lyu, 2023; Xia et al., 2023).

While promising, the reliability and maintainability of machine-generated tests and debugging strategies need further improvement (Ribeiro et al., 2023; Wei et al., 2023). Generating tests that reveal edge cases and complex scenarios is an open challenge, and developers will need techniques to validate and refine the generated tests and fixes.

**Software maintenance and evolution** phase involves modifying and updating software after its initial release. Research indicates AI's potential to revolutionize this area by automating documentation updates (Khan and Uddin, 2022), identifying refactoring opportunities (Rodriguez et al., 2023), and aiding system migration (Su et al., 2023). AI analysis of code repositories could transform maintenance planning and risk assessment by revealing important trends, anomalies, and undocumented dependencies (Le and Zhang, 2023). However, AI's effectiveness often depends on precise input prompts (Le and Zhang, 2023; Rodriguez et al., 2023), and consistent performance across different software environments remains challenging (Su et al., 2023). Advances in AI knowledge representations and reasoning capabilities are essential if AI is to play a larger role in guiding software evolution (Rodriguez et al., 2023).

**Software management** phase is concerned with activities related to overseeing and coordinating the entire development process, including planning, resource allocation, scheduling, and ensuring that a software project meets its goals and deadlines. Research indicates that AI has significant potential to transform software management practices. AI can enhance critical management activities such as project planning (Dam et al., 2019), effort estimation (Hefny et al., 2021), risk assessment, and team coordination (Matthies et al., 2019), potentially leading to more efficient and data-driven decision-making processes. The analysis of project repositories and development metrics by AI systems could provide unprecedented insights into project dynamics and team performance (Voria et al., 2022). Conversational AI agents show promise as virtual project assistants, offering real-time project updates and early issue detection (Matthies et al., 2019). AI-driven task assignment and scheduling based on developer expertise and workloads could optimize resource allocation (Dam et al., 2019). Importantly, the effectiveness of AI in software management hinges on its ability to model complex human factors such as team dynamics, personalities, and sentiment (Voria et al., 2022). The successful integration of AI management assistants is highly dependent on their adaptation to specific organizational cultures and contexts, as evidenced by research into chatbots designed for software engineering teams (Hefny et al., 2021).

**Cross-cutting themes** include the need for high-quality, representative training datasets, human-centered interaction techniques, feedback loops where LLMs learn from developer actions, and the potential to enhance LLM reasoning with domain-specific knowledge bases and program analyzes. The ethical implications of LLMs also deserve careful attention as they increasingly influence the future of software engineering work, education, and research.

### 5.2 RQ2—conversational AI in computing education

**Figure 11:**
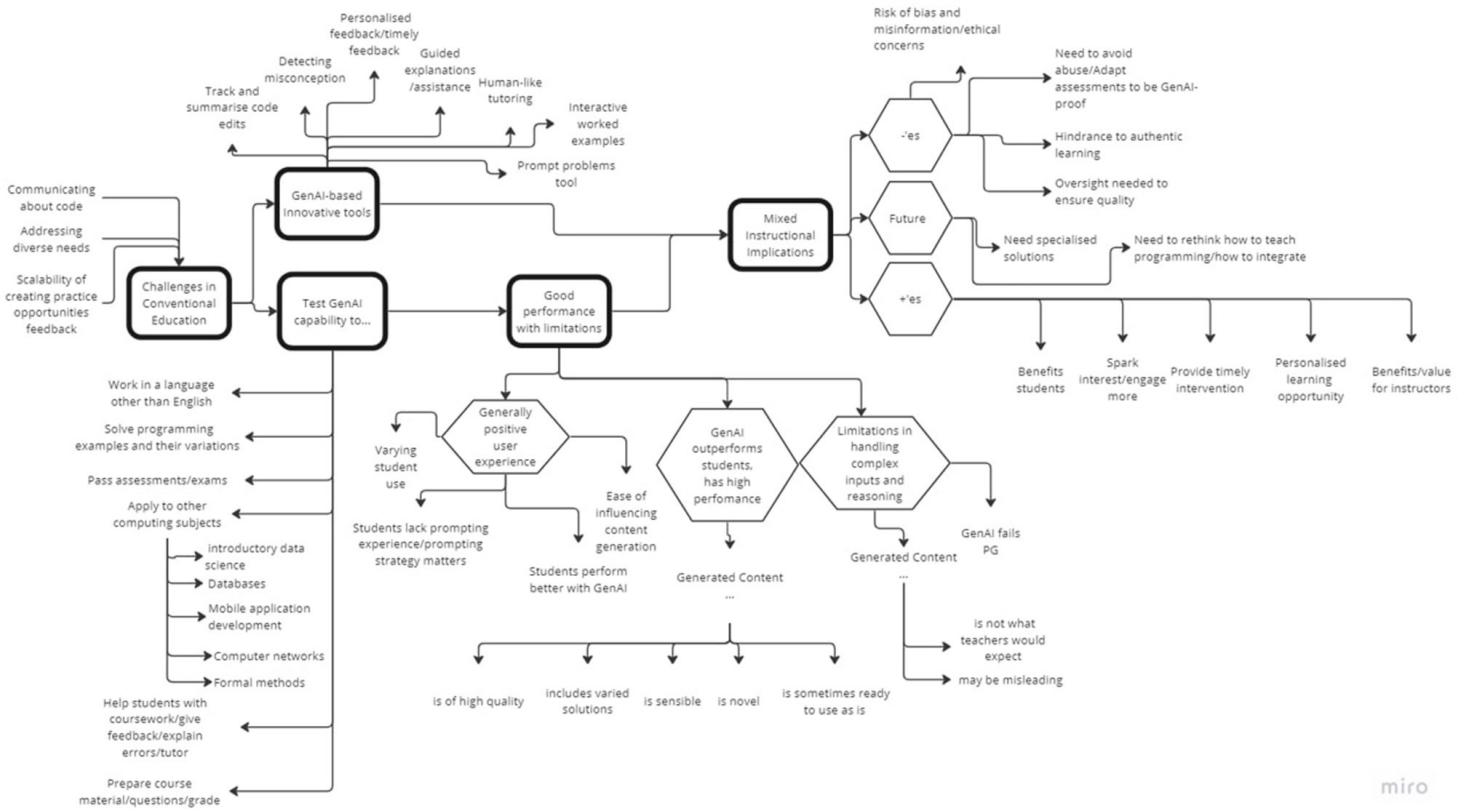
Thematic map, which resulted from reflexive analysis based on CS_HE abstracts.

The analysis of 52 papers under the CS_HE category revealed five key themes: (1) challenges to conventional instruction, (2) testing CAI capabilities, (3) GenAI-based innovative tools, (4) good performance with limitations, and (5) mixed instructional implications, as shown in the Thematic Map in Figure 11. Appendix Table 3 presents the themes, sub-themes, codes, and examples from the abstracts. The first theme, *challenges to conventional instruction*, characterizes the main research motivation for selected abstracts. As a result, the research either explored *testing Conversational AI capabilities* or creating *GenAI-based innovative tools*. The experiments with current tools (e.g., ChatGPT) generally reported *good performance with limitations*. These assessments typically led to *mixed instructional implications*. Collectively, these themes paint a picture of the way how GenAI tools are received in education environments, highlighting their potential as well as limitations.

**Challenges to conventional instruction:** Challenges to conventional instruction served expectedly as a key motivator for current research on GenAI-based instruction. These challenges stemmed from the difficulty in “communicating about code” and “addressing diverse needs,” which also overlaps with the challenge of “scalability of creating practice opportunities and feedback” (see Appendix Table 4) Communication about code is found challenging for both the instructors and students as they struggle with providing code explanations (Oney et al., 2018; Leinonen et al., 2023a,b). While instructors acknowledge the benefits of providing more practice opportunities, having to create and update them constantly is considered time-intensive (Nguyen et al., 2022; Jury et al., 2024). Diverse student needs (Shaka et al., 2023), and large classes are also significant barriers, e.g., to effectively closing the feedback loop (Wang T. et al., 2023) and providing personalized assistance (Sheese et al., 2024).

**Testing conversational AI capabilities:** In our research, we observe a general curiosity about the capabilities of widely popular and available conversational AI like ChatGPT or CoPilot. Most research aims to understand the capabilities of these tools to solve programming examples and their variations (Finnie-Ansley et al., 2022; Berrezueta-Guzman and Krusche, 2023; Wermelinger, 2023). User studies with students, e.g., surveys (Budhiraja et al., 2024) or observations of student use (Prasad et al., 2023; Prather et al., 2023b; Kazemitabaar et al., 2024), aimed to understand how current tools may help student learning or tutor students. These tools were also found helpful for instructors, e.g., in preparing course materials, grading, and feedback (Balse et al., 2023; MacNeil et al., 2023; Speth et al., 2023; Doughty et al., 2024). Our analysis shows that while not explored by many, the research started looking into the capabilities of these tools in instructing or aiding learning subjects other than introductory programming (Nguyen et al., 2022; Lauren and Watta, 2023) and in languages other than English (Cipriano and Alves, 2023). However, there is also a significant concern of misuse and plagiarism, which in turn led to questioning the reliability and validity of current assessment approaches, e.g., testing whether ChatGPT can pass assessments or exams (Dobslaw and Bergh, 2023; Finnie-Ansley et al., 2023; Savelka et al., 2023).

**GenAI-based innovative tools:** One of the key differences between academia and industry is that GenAI is not considered just as a productivity tool for students but also as a pedagogical one (Johnson, 2024). Learning opportunities are expected to improve through personalized support (Shaka et al., 2023) and feedback for students just-in-time (Roest et al., 2024) and 24/7 through virtual one-to-one human-like tutoring (Jell et al., 2023). Personalized support is possible for tracking and summarizing code edits (Oney et al., 2018), detecting misconceptions (Jell et al., 2023), generating next-step suggestions (Roest et al., 2024). Self-sufficiency can further be encouraged by a new concept of prompt problems (Denny et al., 2024). While most of these innovations are centered around improving students' learning experience, there is also potential for improving instructor experience: Jury et al. (2024) explores the use of LLMS for creating interactive worked examples as a way to help instructors scale to large classes.

**Good performance with limitations:** GenAI performance has so far been impressive: solving problems at the first attempt, or with some changes (Denny et al., 2023) and the conversational AI doing well in exams and outperforming most students (Finnie-Ansley et al., 2022). Users also report positive experiences finding the current tools easy to use (Sarsa et al., 2022) even though they may lack prompting experience (Hanifi et al., 2023). Students using the conversational AI tools also perform better (Qureshi, 2023). However, limitations have also been identified in handling complex input and reasoning when the tools are presented with non-textual descriptions or directly program files (Ouh et al., 2023) or are required to handle exercises with a complex chain of reasoning (Savelka et al., 2023). However, with the pace these tools have been evolving, it is not clear how long these limitations will continue to exist.

**Mixed instructional implications:** Regardless of the impressive performance of GenAI-based tools, their reception in educational environments is somewhat mixed (Lau and Guo, 2023). While there is optimism around benefitting students and instructors in providing personalized learning (Mirhosseini et al., 2023; Qadir, 2023; Roest et al., 2024) and timely interventions to smooth the learning journey (Shaka et al., 2023; Sheese et al., 2024), there are significant concerns around ethical use (Prather et al., 2023a; Qadir, 2023), and especially plagiarism and academic dishonesty (Morsy et al., 2023; Rajabi et al., 2023; Richards et al., 2024). While tools to detect AI-generated work may serve as a countermeasure (Morsy et al., 2023), there is a deeper need to rethink teaching and assessment more fundamentally (Dobslaw and Bergh, 2023; Graven and MacKinnon, 2023; Jacques, 2023). There is also a growing concern of over-reliance (Kazemitabaar et al., 2023; Randall et al., 2023), e.g., when conversational agents lack current training data and, hence, cannot answer queries, and may mislead students (Balse et al., 2023; Hanifi et al., 2023; Roest et al., 2024). Therefore, oversight is needed to ensure quality (Sarsa et al., 2022; Speth et al., 2023). Furthermore, more specialized solutions may be needed for computing subjects other than introductory programming (Ahmed and Hasnine, 2023). Despite these concerns, the way forward is working toward a better-guided integration to computing classrooms (Chen E. et al., 2023; Graven and MacKinnon, 2023; Liu, 2023; Rajabi et al., 2023; Randall et al., 2023).

### 5.3 RQ3—early experience and promising improvements in software engineering education

Finally, in this section, we have sought to understand the ways and processes of introducing LLM-based conversational agents specifically in software engineering curricula, expanding on what we learned from the previous sections. The analysis in this section was based on the full text of 16 papers. The Table 2 summarizes each paper, showing studies covered a range of software engineering topics (detailed below) and varying levels of maturity ranging from basic experimentation with a CAI to a proof-of-concept to pilot studies run with 10–100 students.

**Table 2:** Analysis results of papers that focus on conversational agents in software engineering education.

**References**	**SE area**	**Target group**	**Conversational agent**	**Study type**
Dehbozorgi and Norkham (2021)	Modeling	Instructors	Conversational recommender system for recommending design patterns	Proof-of-concept
Paschoal et al. (2018)	Requirements	UG students in SE	A CA, Ubibot, provides personalized interactions and simulates real-world stakeholder scenarios	Pilot study with 15–30 students
Gorer and Aydemir (2023)	Requirements	Educators	GPT-3.5 and Bard to generate requirements interviews with typical analysts mistakes for interview training	Proof-of-concept
Abdelfattah et al. (2023)	Requirements Modeling	Students in SE	ChatGPT in dialog to generate e.g., user stories, use case diagrams, class diagrams, and sequence diagrams	Proof-of-concept
Ren et al. (2023)	Modeling	CS and ES students	A CA, SOCIO, for UML modeling tasks	Controlled experiments with 132 students in 44 teams
Ciupe et al. (2019)	Agile (Scrum)	UG students in Applied Informatics	A CA with structured knowledgebase	Pilot study with 200 students
González et al. (2022)	Agile	UG students	A custom CA for providing support in SE Capstone projects	Controlled experiment with 131 students
Valový and Buchalcevova (2023)	Agile (Pair Programming)	No specific group	ChatGPT and Github CoPilot	Controlled experiments with 38 students; interviews with 5
Manfredi et al. (2023)	Agile (Pair programming)	UG Computing students	MR application using HoloLens and a conversational virtual avatar, interacting with users through an integrated CA	Controlled experiments with students; post-assessment survey
Jalil et al. (2023)	Testing	Students and instructors	ChatGPT tested on first five chapters of a software testing textbook by Ammann and Offutt (CITE)	Experiment with CAI
Paschoal et al. (2023)	Testing	UG students in Information Systems	A custom CA, TOB-STT, for providing educational support in software testing	Controlled experiment with 38 students
Farah et al. (2022a)	Code Reviews	Students in SE tertiary education	A dialog-based CA, based on a code review application designed to teach programming best practices	Proof-of-concept
Farah et al. (2022b)	Code Reviews	UG Students in SE	Instructors impersonate a CA, LintBot, embedded in an online learning application that simulates the code review features of social coding platforms	Controlled in-class experiment with 30 students
Bull and Kharrufa (2023)	Code Reviews	Instructors	GenAI in general including Github CoPilot and ChatGPT	Interviews with 5 professional developers
Li J. et al. (2023)	Security	UG students	GPT-4 tested on a security coursework	Experiment with CAI
Reynolds et al. (2023)	General	Educators	ChatGPT, BERT used to link assessment to learning outcomes in SE	Experiment with CAI

The rest of the section discusses findings based on the following SE areas: Requirements engineering, software design, software management, software quality assurance, and software security. Unlike the software engineering practice analysis in Section 5.1, the software maintenance and evolution area is not represented. On the other hand, software development-related work is mostly concerned with introductory and seldom intermediate programming and is presented in 5.2; however, we included one paper on secure software development.

**Requirements engineering:** One of the most used techniques for requirements extraction is interviewing stakeholders, and the papers included in our study describe ways to support the training of software engineering students in this area. In 2018, Paschoal et al. (2018) explored the development and evaluation of a context-sensitive chatterbot named Ubibot, designed to enhance software engineering education by focusing on requirements extraction and assisting students in creating requirements documentation. By adapting to the user's knowledge level and performance, Ubibot offered tailored support, aiming to bridge the gap in practical skills among computing students. Through a pilot study with undergraduate students and an experimental comparison with a non-context-sensitive version, the authors found that Ubibot effectively supports learning by providing personalized interactions and simulating real-world stakeholder scenarios.

In 2023, Gorer and Aydemir (2023)'s study questions whether such requirements engineering support is also possible with the current generative AI tools, i.e., not specifically built on a knowledge base like the Ubibot (Paschoal et al., 2018). Hence, the authors apply prompt engineering to GPT-3.5 and Bard to generate interview scripts incorporating typical analyst mistakes as educational material for interview training. The preliminary evaluation focuses on three different products, each in distinct business domains: call center software, digital health platform, and project management software. Employing a batch generation approach, four interview scripts for each case were produced—three containing intentional mistakes and one error-free. Then, the authors considered the iterative generation, and the initial few turns of a complete interview script were used to generate subsequent turns with intended mistakes. The authors faced challenges in achieving satisfactory performance for generating certain mistake types, such as “Not asking for existing system,” “Ignoring other stakeholders,” and “Asking long questions.” ChatGPT outperformed Bard in terms of generating more complete and coherent interview scripts for the given product features in the case of batch generation. However, Bard excelled in the iterative generation, generating more accurate interview turns for specific mistake types and providing more formal explanations of how the mistakes were incorporated into the analyst's question. The study concludes LLMs require domain-specific training to improve quality. There is much to explore, as the generated interviews were not evaluated by experts in terms of quality, and they have not been tested with instructors and students in terms of their utility as a training tool.

Similarly, Abdelfattah et al. (2023) consider instructing students on the requirements of engineering principles via interactive exercises and hands-on examples through ChatGPT. The paper proposes a student-ChatGPT conversation flow to generate various software engineering artifacts, starting from user stories, escalating to use case diagrams and class diagrams, and culminating with sequence diagrams, which go into software design and are discussed in the next section. However, while this work imagines how ChatGPT could be helpful in teaching software engineering concepts to students, one question that arises is whether the students would need to be already well-versed in software engineering to be able to prompt ChatGPT as described in this work, creating a “Chicken-Egg problem.”

**Software design and modeling:** UML use cases, class diagrams, and sequence diagrams are valuable yet challenging concepts for students. These topics are often introduced early in the curriculum when students may not have fully developed their coding skills. Several studies in software engineering practice have explored the use of LLMs to support various aspects of the software design process, such as enhancing the quality of UML use case scenarios, automating domain modeling, and assisting in UML diagram generation (Cámara et al., 2023; Cámara et al., 2024; Chen K. et al., 2023; De Vito et al., 2023).

In software engineering education, Ren et al. (2023) presents a study on the effectiveness of the SOCIO chatbot in facilitating UML modeling tasks compared to a conventional online web tool, Creately.^4^ Conducted in an academic setting, this research is centered around evaluating the usability of SOCIO by comparing its efficiency, effectiveness, satisfaction levels among students, and the quality of class diagrams produced with those created using Creately. Through a series of controlled experiments, the study finds that students were generally faster at building class diagrams and more satisfied with their experience when using the SOCIO chatbot. Although the diagrams produced via SOCIO tended to be more concise, they were slightly less complete compared to those generated with Creately. Nevertheless, the findings suggest that chatbots like SOCIO can be valuable tools for UML modeling. Future research should aim to enhance LLM capabilities in understanding domain semantics, generating more complete and consistent models, adhering to established modeling practices, and effectively supporting student learning to maximize their potential as tools in software engineering education.

**Secure software development:** While more basic software development work was covered in Section 5.2, we have included one paper that explored the potential of ChatGPT-4 in enhancing secure software development education in an undergraduate software security course (Li J. et al., 2023). Teaching assistants evaluated the model's performance on exercises involving code from a web application used by 200 students, specifically testing its ability to detect vulnerabilities, suggest penetration testing strategies, and propose fixes. The results were promising, with ChatGPT-4 identifying 20 out of 28 vulnerabilities while also reporting three false positives and detecting four additional vulnerabilities not originally identified. This indicates not only the capability of LLMs like ChatGPT-4 to assist in educational settings but also the need to update course exercise design and grading systems in the era of LLMs.

**Software management:** The papers in this category mainly focus on Agile Methods. Agile methods were born in the 1990's from the need to reduce the overhead of heavyweight, plan-based methods used in large-scale software development projects (Bourque and Fairley, 2014). In 2019, Ciupe et al. (2019) created a structured knowledge base for a chatbot to facilitate learning of Agile Scrum concepts. The chatbot system integrated learning analytics to monitor and analyze student interactions. In an experimental assessment with 200 students, the students interacted with two versions of the chatbot, one providing guidance through clues and another without, to explore the chatbot's effectiveness. The authors tracked the complexity of the questions asked, the interaction patterns, and the progression through the learning material to assess the chatbot's impact on the students' learning experience. They observed that students engaged more deeply with the material and reached higher complexity levels of understanding when interacting with the chatbot version that provided clues and guided learning. The results indicate the importance of personalizing the learning experience by adapting the chatbot interactions to the learner's progress and preferences. The study considered the chatbot as an effective complement to traditional teaching methods or within a flipped classroom model. González et al. (2022)'s work also confirms the utility of virtual assistants. By training a virtual assistant based on the lessons learned by past students, the students were able to better understand the management of their software engineering capstone projects.

Valový and Buchalcevova (2023) focused on AI-assisted pair programming, a method frequently used in Agile software development (Bourque and Fairley, 2014). The authors ran seven experimental programming sessions on an undergraduate student sample (38 students from two classrooms) in applied informatics courses, subjecting them to solo, pair, AI-assisted settings using ChatGPT and Github CoPilot. They also ran a qualitative study based on five semi-structured interviews with participants, and five with professionals who have five or more years of experience and had exposure to AI tools in their jobs. The interview explored familiarity with AI, the applicability of AI in various tasks, AI's personality and emotions, psychological aspects of AI, effectiveness and efficiency, and future prospects. In their study, both professionals and students find AI tools beneficial for improving effectiveness and efficiency in programming tasks, resulting in faster development, better quality of work, and fewer bugs. These participants were also abandoning tools like Google Search or Stack Overflow and used AI to take care of mundane tasks like writing tests and documenting code. This move away from Stack Overflow was also reported in Burtch et al. (2024); however, repeating their analysis on Reddit communities that focus on the same sets of technology topics, virtually no evidence of any decline in participation following ChatGPT's emergence was found. So, “a robust social fabric” may assure the health and sustainability of online knowledge communities going forward.

In Valový and Buchalcevova (2023), participants would prefer a human to pair with, with the exception of self-described “introverts.” Perhaps unsurprisingly, in this study, participants also attributed all success to themselves and all failures to AI, i.e., without them, AI would be “useless.” They also found AI too apologetic. These perceptions are somewhat in contrast to the reception of the mixed-reality virtual assistant, created using the HoloLens device that incorporates a conversational virtual avatar (Manfredi et al., 2023). The avatar interacts with the user and provides suggestions as students code in two modes—a driver (CAI generates code) or a navigator (CAI suggests code improvements). The preliminary evaluation in a real teaching scenario with students showed that students using the virtual assistant had statistically significant improvements in coding skills compared to the control group in a traditional pair-programming setting. The surveys indicated a high user satisfaction with the features, usability, and effectiveness of the virtual assistant in addressing the challenges of finding and collaborating with a human partner.

AI is indeed causing a programming paradigm shift from descriptive to declarative, learning software engineering by reverse engineering code. “The Chicken-Egg Problem” is also apparent here as students in Valový and Buchalcevova (2023) struggled with explaining themselves to AI and wished “AI could read their minds.” Students in these studies often expressed that the basics of software engineering must be taught without AI and before AI-assisted programming. On the other hand, professionals in their study were comfortable with expressing themselves clearly to AI.

**Software quality:** Some preliminary efforts to integrate chatbots in the area of software quality focus on software testing and code reviews. In the area of software testing, Paschoal et al. (2023) evaluate the capabilities of a custom conversational agent, TOB-STT, to support testing activities and measures students' capabilities for bug identification with and without using the tool. The study showed no significant improvement in students' defect identification skills, likely due to the inadequate capabilities of the custom CA. Jalil et al. (2023) examine the effectiveness of ChatGPT in answering practice questions from standard software testing curricula. This study demonstrated that ChatGPT, when engaged in a shared conversational context, more frequently provided correct or partially correct responses compared to separate conversational contexts. ChatGPT's self-reported confidence did not correlate strongly with the accuracy of its answers, indicating a need for calibration improvements. Despite these challenges, LLMs are found to have the potential to clarify complex topics in software testing education.

In the area of code reviews, Farah et al. (2022b) designed a code review application to teach programming best practices, aiming to provide a blueprint of interactions between a student and a conventional chatbot. The authors show how a student can interact with a chatbot, which, in their example, mainly provides code formatting and variable naming suggestions. In Farah et al. (2022a), an online learning application was proposed to simulate the code review features available on social coding platforms and allow instructors to interact with students using a chatbot identity, LintBot. The goal of this application is to increase student engagement during code review exercises while also reducing instructors' workloads with respect to the guidance required during these exercises. The controlled in-class experiment comprising 30 undergraduate software engineering students evaluated two treatments: (i) providing explanations as the course instructor within the code review application (instructor condition) and (ii) providing explanations within the code review application through a chatbot that the course instructor impersonated (chatbot condition). Here, LintBot was simply an identity and did not have any agency or script to reply automatically to student comments, and students assigned to this condition were unaware that the instructor impersonated the chatbot. While the high SUS (System Usability Scale) scores for the application were promising, the authors found no significant differences across the different groups in terms of learning, while engagement was higher with the instructor, even though not significantly. The lack of difference was attributed to the repetitiveness of examples, and they considered the mere use of identities (human or chatbot), to present static information, to add very little to the learning experience.

Current LLM-based tools can easily replace these early studies. For example, we have run the same experiment (Figure 5; Farah et al., 2022b) with ChatGPT-GPT4o to see how much LLM-based agents provide this functionality out of the box. We received more advanced feedback about variable naming and the use of data structures, and ChatGPT also provided an alternate, cleaner version of the code. When asked to tune the response for a person new to programming, ChatGPT also modified the response to provide a simplified version, a step-by-step explanation, and a highlight of concepts for beginners.

Bull and Kharrufa (2023) considered current GenAI tools as promising to include “code reviews” as a common practice early on in software engineering education. Nevertheless, there is still further research needed to explore the impact of instructor-generated feedback vs automated feedback on code-review exercises.

## 6 Discussion and future directions

We started this study with the goal of examining the current state and future directions of conversational AI in software engineering. Using a rapid review approach guided by the PRISMA method, we selected 183 relevant peer-reviewed articles. The quantitative analysis revealed a significant increase in publications on conversational AI in software engineering and computing education from 2018 to 2023, spanning numerous countries with a few large collaborative communities and many smaller, isolated groups, all indicating a growing interest in this area.

We aimed to answer three main research questions through a qualitative analysis of the papers included in this study:

- **RQ1: How is conversational AI currently influencing the software engineering industry?**
  Conversational AI has the potential to disrupt various phases of the software development lifecycle (SDLC). While much of the current research focuses on code generation and repair, conversational AI is also being integrated into team training, expert recommendations, documentation, and maintenance tasks. The proficiency of applying conversational AI varies across different SDLC areas, but the general trend is toward integrating AI while maintaining human oversight to mitigate limitations. The key challenge lies in increasing developers' awareness of both the potential and limitations of conversational AI tools, as the technology is still evolving, and its usage is not fully understood.
- **RQ2: How is conversational AI impacting computing education?**
  Conversational AI is transforming computing education by providing automated feedback, personalized learning experiences, and interactive worked examples. The adoption of AI-driven tools in education has shown promise, particularly in introductory programming courses, but there are also significant challenges, such as academic integrity concerns and the need for effective assessment methods. Empirical studies indicate that while AI tools can enhance learning, provide real-time feedback and support, helping students understand complex concepts and complete assignments more efficiently, their limitations in handling complex reasoning and non-textual descriptions must be addressed.
- **RQ3: What do early experiences show in terms of promising improvements in educating future software engineers?**
  Early experiences suggest that integrating conversational AI into software engineering education can improve student engagement and learning experience. However, the work is very preliminary, and there is a need for more specialized CAI and further empirical studies to evaluate the long-term impact of these tools on student performance and to develop best practices for their use. Additionally, addressing the “Chicken-Egg” problem, where effective use of AI tools requires pre-existing software engineering knowledge, is crucial for maximizing their educational benefits.

Based on these findings, future research should focus on creating AI-driven educational tools and teaching methods evolving from current basic programming to support the learning of more advanced concepts. In software engineering practice, the emphasis on prompt engineering shows the need for clear guidelines and best practices for using conversational AI in various tasks. Researchers and industry professionals should collaborate to develop and standardize effective prompts across different areas of software engineering.

However, there is a risk that students might over-trust these tools and use them without critical evaluation. Such reliance can result in a superficial grasp of concepts and inadequate problem-solving skills. To mitigate this risk, educators must highlight the importance of using conversational AI as an aid rather than a replacement, i.e., “coPilot, not auto-pilot.” While the lack of originality might be insignificant for routine code generation, it is fundamental for creative problem-solving in software engineering. The challenge is to turn conversational AI into a partner in software innovation and product creation, particularly as hybrid teams of humans and AI become more common. Educators should focus on integrating AI in a way that promotes critical thinking and a deeper understanding of the material, ensuring that AI supports rather than supplants student effort. This approach will help ensure that the use of AI in education promotes genuine learning and the development of essential skills.

The adoption of ChatGPT and similar tools also carries certain risks related to the propagation of incorrect information and issues related to academic integrity. To mitigate these risks, the use of AI tools should be considered within the framework of the Artificial Intelligence Risk Management Framework (AI RMF 1.0; Tabassi, 2023), which provides guidelines for assessing and managing the risks associated with AI technologies, ensuring their safe and ethical use. The AI RMF emphasizes the importance of transparency, accountability, and fairness in AI deployment. Applying this framework in educational contexts involves implementing measures to ensure that AI tools are used responsibly. This includes training educators and students on the appropriate use of AI, establishing protocols for verifying the accuracy of AI-provided information and developing strategies to detect and prevent academic misconduct facilitated by AI.

While we identify several promising areas for integrating conversational AI in software engineering education, such as requirements elicitation, software modeling, software management, and software quality, more empirical studies are needed to assess the impact of these interventions on student learning. Personalized learning can tailor educational experiences to individual student needs, potentially increasing motivation and improving comprehension. However, the effectiveness of these AI-driven educational tools must be rigorously evaluated. It is vital to conduct empirical studies to understand how these tools influence various aspects of learning, such as knowledge retention, problem-solving skills, and student satisfaction.

In this context, several open questions require further investigation:

- What are the implications of reversing the learning process—producing software to understand concepts rather than understanding concepts to produce software—on creativity, problem-solving abilities, and decision-making?
- How do different student populations, including those from diverse educational backgrounds and varying levels of prior knowledge, benefit from CAI? How can we address issues of access and equity in education concerning AI-driven learning tools?
- Is social interaction still crucial, or can conversational agents replace group work in computer science education? How will AI-human interaction change human-human interaction within software teams?
- How are tasks allocated between humans and AI in such partnerships? Will human participants feel the same sense of achievement?

In summary, future research directions should focus on the long-term effects of conversational AI on student learning, the development of AI-driven tools for collaborative software engineering, and the ethical implications of AI in software development and education.

## 7 Conclusions

With COVID-19, the education sector has already seen significant disruption and permanent changes to learning, teaching, and assessment, and the technological shift will continue with generative AI platforms. However, it is still too early to assess the impact of conversational AI technologies when public opinion on inaccurate content and sources and lack of originality shapes. Weighing pros and cons, experts still question the use of generative AI, especially in education and research—“a flawed technology in a flawed society” (Dwivedi et al., 2023). Nevertheless, the studies in our review almost unanimously agree that students and educators must learn to work with conversational AI to help prepare for the future.

Specifically within Software Engineering, the increasing capabilities of AI, particularly LLMs, are beginning to transform tools and practices across the software development lifecycle. From engaging with stakeholders to generating code and tests to providing maintenance recommendations, AI assistants are demonstrating the potential to significantly boost the productivity of software engineers. However, fully realizing this potential requires further advances in the reliability, interpretability, and adaptability of AI models. Even with these advances, human judgment and creativity will remain vital, and AI is best viewed as an augmentation to human developers rather than a replacement.

Given the speed at which automated software engineering is evolving and improving and how rapidly software engineering roles are changing in the industry, there is an urgency for higher education to evolve quickly (Johnson, 2024). In the fast-paced technology landscape, software engineering education should focus on teaching students to keep up with the changes, developing a growth mindset, and using AI assistants while being critical of their limitations—building the skills to effectively guide, interpret, and validate their outputs. Software engineering students need to understand that software projects are not always easy to specify, and software requirements may change over time, as software creation is a more “social endeavor” that evolves with the interactions among the development team, customers, and other stakeholders (Yellin, 2023). Hence, as AI matures, finding the right balance and interface between human and artificial intelligence in the software development process will be key to maximizing their combined potential.

## Author contributions

CS: Conceptualization, Methodology, Software, Visualization, Writing – original draft, Writing – review & editing. RN: Methodology, Software, Visualization, Writing – original draft, Writing – review & editing. GD: Methodology, Validation, Writing – original draft, Writing – review & editing.

## Footnotes

^1^Google (2023). *Gemini*. Available online at: https://gemini.google.com/.

^2^Anthropic (2023). *Claude*. Available online at: https://www.anthropic.com/claude.

^3^See Appendix Table 2 for the full table with references.

^4^ https://creately.com/
